# Low calf circumference can predict nutritional risk and mortality in adults with metabolic syndrome aged over 80 years

**DOI:** 10.1186/s12902-022-00964-1

**Published:** 2022-02-23

**Authors:** Chenxi Ren, Xiaoyan Zhang, Yunxia Zhu, Jun Xu, Ying Xie

**Affiliations:** 1 Department of Endocrinology, The Second Affiliated Hospital of Soochow University, Sanxiang Road, 1055, Gusu District, Suzhou City, Jiangsu Province People’s Republic of China; 2 Department of Geriatrics, Shanghai Jiaotong University Affiliated Sixth People’s Hospital, Shanghai, 200233 People’s Republic of China

**Keywords:** Calf circumference, Nutritional risk, Metabolic syndrome, NRS 2002, Mortality

## Abstract

**Background:**

Metabolic disorders and malnutrition are a double burden worldwide. The aim was to determine whether low calf circumference (CC) could predict nutritional risk and the cut-off values of CC for predicting nutritional risk in metabolic syndrome (MetS) patients aged over 80 years. We aimed to evaluate the risk factors for predicting mortality in MetS.

**Methods:**

A total of 514 patients aged over 80 years with MetS were enrolled and followed for 2.5 years. On admission, demographic data, CC, and laboratory parameters were obtained. Patients with a Nutritional Risk Screening 2002 (NRS 2002) total score ≥ 3 were considered to have nutritional risk.

**Results:**

The CC level was significantly lower in the nutritional risk group than in the non-nutritional risk with MetS group (27.1 ± 4.0 cm vs. 30.8 ± 3.9 cm). Logistic regression analysis of nutritional risk revealed that increasing CC (adjusted OR, 0.81; 95% CI, 0.74–0.88) was an independent protective factor against nutrition risk. The best CC cut-off value for predicting nutritional risk according to the NRS 2002 was 28.8 cm. Cox regression multivariate models showed nutritional risk (HR, 2.48; 95% CI, 1.22–5.04) and decreased CC (HR, 2.78; 95% CI, 1.27–5.98) remained independent risk factors for mortality.

**Conclusion:**

Decreased CC could predict not only nutritional risk but also mortality in MetS patients aged over 80 years. The elderly who had MetS with nutritional risk should be discovered early, early intervention and early treatment. CC may be a valuable index to screen out this population.

## Background

With the rapid development of science and technology, the problem of ageing is becoming increasingly serious. The proportion of adults over 65 to adults of working age in most parts of the world is expected to almost double in the next 40 years [1]. Moreover, the very old people group (adults aged more than 80) has rapidly risen in the last century [2]. As people enjoy longevity, the prevalence of metabolic disorders and malnutrition is significantly increasing and has become a double burden worldwide [3].

Metabolic syndrome (MetS) is a cluster of multiple metabolic abnormalities, including abdominal obesity, dyslipidaemia, elevated blood pressure, insulin resistance, and hyperglycaemia. MetS is associated with a 2-fold increase in cardiovascular disease risk, cardiovascular mortality, myocardial infarction, and stroke, and a 1.5-fold increase in all-cause mortality [4, 5]. According to the International Diabetes Federation (IDF) report, people aged over 65 years are particularly liable to have MetS, and the prevalence of MetS ranges from 37 to 41.9% [6]. Even recently, the Singapore Population Health Study reported that the prevalence of MetS is 50.0% in older adults aged over 85 years [7].

On the other hand, increasing disease-associated malnutrition or nutritional risk, is also an important issue that can cause a significant economic burden [8]. Some research has shown that malnutrition or nutritional risk is associated with a high incidence rate of long hospital stays and high mortality [8, 9]. Some studies have identified that diabetes [10], obesity [11], and hypertension [12] are associated with malnutrition or nutritional risk increases in both young and old people. As a cluster of multiple metabolic abnormalities, the nutritional risk of MetS has not received much attention. Kim HJ identified that an increased nutritional risk was related to elderly MetS patients [13]. Furthermore, MetS with malnutrition causes high mortality in chronic kidney patients [14]. Since MetS with high nutritional risk patients may have a worse prognosis, it is necessary to confirm high-risk individuals and provide appropriate interventions in this population.

Calf circumference (CC) is a stable, highly accessible, and easily measurable index that is better than)body mass index (BMI) or waist circumference (WC) in reflecting muscle loss of the lower extremities with ageing or decreasing physical activity. In some studies, CC has been shown to be a screening method to diagnose sarcopenia, nutritional status, and mortality in hospitalized patients [15–18]. A study identified that CC could be simply substituted for WC and showed higher all-cause and cancer mortality risks than the traditional definition of MetS [19]. Since the measurement of nutritional status in elderly people may serve as a marker for MetS, CC may have the potential to identify MetS with nutritional risk. Our previous study identified that CC could predict nutritional risk in elderly people [15]. However, there is a lack of evidence to identify the association between CC and nutritional risk in the MetS group. In addition, whether CC or other factors could predict mortality is unknown in MetS.

Here, in the above statement, the aim of this study was to determine whether CC could predict nutritional risk in MetS patients aged over 80 years. If CC was an important factor in predicting nutritional status in our study, we identified the cut-off value of CC. More importantly, we further aimed to evaluate the risk factors for predicting mortality in MetS.

## Methods

### Study population

A total of 1234 hospitalized patients admitted to the Department of Geriatrics at Shanghai Jiaotong University Affiliated Sixth People’s Hospital between July 2017 and April 2018 were recruited for this study. We included people who were diagnosed with MetS according to the American National Cholesterol Education Programme (Adult Treatment Panel III) guidelines [20] for more than 80 years and who had complete medical and nutritional information available from the medical records of the institution. We excluded people who were non-MetS, were less than 80 years, had presence of carcinomatous cachexia (referent to the clinical history), had critical illness, were unable to communicate, had bedridden status, or had oedema. Finally, 514 hospitalized participants were included in the analysis and followed up by October 30, 2019. Due to the participants accepting healthcare at Shanghai Jiaotong University Affiliated Sixth People’s Hospital, there were no missing follow-ups. The study was approved by the Ethics Committee of the Shanghai Jiaotong University Affiliated Sixth People’s Hospital [approval number, 2016–141-(1)]. Written informed consent was obtained from all participants and in accordance with the principle of the Helsinki Declaration. Written informed consent was obtained from each participant.

### Data collection

Detailed information about medical history and lifestyle, including smoking and drinking status, was obtained using questionnaires and confirmed through examination of medical records by trained physicians. Current smoking status was defined as yes if the subject smoked at least one cigarette per day or seven cigarettes per week in the past 6 months. Current drinking status was defined as yes if the subject consumed alcohol at least once a week in the past 6 months. Blood pressure (BP) was measured at the nondominant arm in a seated position after a 5-min rest using an automated electronic device (OMRON Model HEM-752 FUZZY’ Omron Co., Dalian, China). Three measurements were taken one minute apart, and the average of the three measurements was used in the analysis. The diagnostic criteria of MetS and its components - abdominal obesity, diabetes, hypertension, high triglycerides, decreased HDL-c were defined according to the Adult Treatment Panel III guidelines [20]. Coronary heart disease was identified if patients had a myocardial ischaemic history or electrocardiographic typical ischaemia pattern. Cerebral infarction was confirmed as a history of ischaemia attack confirmed by cerebral CT or MRI scan.

### Anthropometric measurement

Body height, weight, and WC were measured by experienced physicians. Height and weight were recorded to the nearest 0.1 cm and 0.1 kg, respectively, while participants were wearing light indoor clothing without shoes. BMI (kg/m^2^) was calculated as weight in kilograms divided by height in square metres. WC was measured to the nearest 0.1 cm with participants in the standing position. The greatest circumference of the lower right leg was measured in the standing position as CC [21].

### Nutritional risk assessment

NRS 2002 was used to determine nutritional risk. The total NRS 2002 score indicates whether the patient is nutritional risk (score ≥ 3) or non-nutritional risk (score < 3) [22]. A multidisciplinary nutrition support team evaluated the nutritional status of each patient. All patients underwent nutritional status assessment in the first 24 h of hospital stay.

### Laboratory measurements

All patients fasted overnight before blood samples were collected. Haemoglobin levels were measured using a standard cyanmethemoglobin method. The measurements of fasting plasma glucose (FPG), triglycerides (TG), total cholesterol (TC), low-density lipoprotein cholesterol (LDL-c), and high-density lipoprotein cholesterol (HDL-c) were serum albumin were assessed using turbidimetric immunoassay (Hitachi, Tokyo, Japan). Haemoglobin levels were measured using a standard cyanmethemoglobin method. Haemoglobin A1c (HbA1c) was measured by high-performance liquid chromatography using Bio-Rad D-10 (Bio-Rad, Inc. USA).

### Statistical analysis

SAS version 9.1 (SAS Institute, Cary, NC) was used for database management and statistical analysis. Data are presented as the means ± standard deviation (SD) for continuous variables or numbers (percentages) for categorical variables. Comparisons of means and proportions were performed with variance analysis and x^2^ tests, respectively. The association between CC and other variables was evaluated with Pearson or Spearman correlation analysis. In addition, we used logistic regression analysis to evaluate the association of CC with nutritional risk. A receiver operating characteristic (ROC) curve was used to identify the optimal CC cut-off point to predict malnutrition. Multivariable Cox regression models with hazard ratios (HRs) and 95% CIs were conducted to examine the association of independent factors with mortality. In the present study, subjects with CC values below 28.8 cm (the optimal CC cut-off point to predict nutritional risk according to the ROC curve) were defined as those with decreased CC. Adjusted odds)ratios (ORs), HRs and corresponding 95% confidence intervals (CIs) were calculated, and *P* <  0.05 was considered statistically significant.

## Results

### General characteristics of the participants

A total of 514 hospitalized patients participated in this study (mean age, 86.5 ± 6.0 years; male/female, 368/146); 43.58% in our patients of advanced age had malnutrition risk. Table 1 shows the nutritional risk and non-nutritional risk subjects’ clinical characteristics. The FPG, albumin, haemoglobin, BMI, WC and prevalence of abdominal obesity were significantly different between the two groups (*P* = 0.02, < 0.01, < 0.01, < 0.01, < 0.01 and <  0.01, respectively). A total of 514 MetS patients were followed for 2.5 years at the longest, 2.0 years on the average, and 105 patients died. The mortality of MetS was higher in the nutritional risk group than in the non-nutritional risk group (35.3% vs. 8.4%, *P* <  0.01). However, age, sex, smoking status, drinking status, systolic BP (SBP), diastolic BP (DBP), HbA1c, TC, TG, LDL-c, and HDL-c were not significantly different between the nutritional risk and non-nutritional risk participants (all *P* > 0.05). The prevalence of cerebral infarction, coronary heart disease, diabetes, hypertension, high triglycerides, and decreased HDL-c were not different between the two groups *(P* = 0.10, *P* = 0.24, *P* = 0.67, *P* = 0.81, *P* = 0.08 and *P* = 0.12, respectively).

**Table 1:** Characteristics of Nutritional risk and Non-Nutritional risk MetS adults

	All included patients (*n* = 514)	Nutritional risk (*n* = 224)	Non-Nutritional risk (*n* = 290)	*P*
Age (years)	86.5 ± 6.0	86.9 ± 5.3	86.2 ± 6.1	0.18
Male n (%)	368 (71.6)	160 (71.4)	208 (74.7)	0.37
Smoking status n (%)	92 (17.9)	42 (18.8)	50 (17.2)	0.80
Drinking status n (%)	11 (2.1)	5 (2.2)	6 (2.1)	0.10
SBP (mm Hg)	132.0 ± 18.9	131.5 ± 18.8	132.4 ± 19.0	0.60
DBP (mm Hg)	71.5 ± 11.3	71.1 ± 11.7	71.0 ± 11.0	0.34
HbA1c (%)	6.4 ± 1.1	6.2 ± 1.3	6.5 ± 1.0	0.16
FPG (mmol/l)^#^	6.2 ± 2.0	6.6 ± 2.3	6.1 ± 1.8	0.02
TC (mmol/L)	4.1 ± 1.1	4.0 ± 1.1	4.1 ± 1.1	0.40
TG (mmol/L)	1.5 ± 0.9	1.5 ± 0.8	1.4 ± 0.9	0.69
LDL-c (mmol/L)	2.3 ± 0.8	2.3 ± 0.8	2.3 ± 0.8	0.98
HDL-c (mmol/L)	1.0 ± 0.3	1.0 ± 0.3	1.0 ± 0.3	0.07
Albumin (g/dL)^#^	37.5 ± 52	35.2 ± 4.7	39.4 ± 5.3	< 0.01
Haemoglobin (g/dL)^#^	114.1 ± 21.4	105.3 ± 21.8	121.5 ± 17.9	< 0.01
BMI (kg/m^2)#^	23.5 ± 3.7	22.1 ± 3.7	24.7 ± 3.2	< 0.01
WC (cm)^#^	90.7 ± 11.0	87.0 ± 12.0	93.7 ± 9.2	< 0.01
Mortality n (%)^#^	105 (20.4)	79 (35.3)	26 (8.4)	< 0.01
Cerebral infarction n (%)	269 (52.3)	129 (57.6)	140 (48.3)	0.10
Coronary heart disease n (%)	347 (67.5)	160 (71.4)	187 (64.5)	0.24
MetS components	___	___	___	___
Abdominal obesity n (%)^#^	296 (57.6)	105 (46.9)	191 (65.9)	< 0.01
Diabetes n (%)	274 (53.3)	117 (52.2)	157 (54.1)	0.67
Hypertension n (%)	471 (91.6)	206 (92.0)	265 (91.4)	0.81
High triglycerides n (%)	93 (18.1)	33 (14.7)	60 (20.7)	0.08
Decreased HDL-c n (%)	246 (47.9)	88 (39.3)	158 (54.5)	0.12

BMI, body mass index; DBP, diastolic blood pressure; FPG, fasting plasma glucose; HbA1c,Haemoglobin A1c; HDL-c, high-density lipoprotein cholesterol; LDL-c, low-density lipoprotein cholesterol; MetS, metabolic syndrome; SBP, systolic blood pressure; TC, total cholesterol; TG, triglyceride; WC, waist circumference

^#^*P* <  0.05 versus nutritional risk and non-nutritional risk group

### The difference in CC between nutritional risk and non-nutritional risk participants

**Fig. 1:**
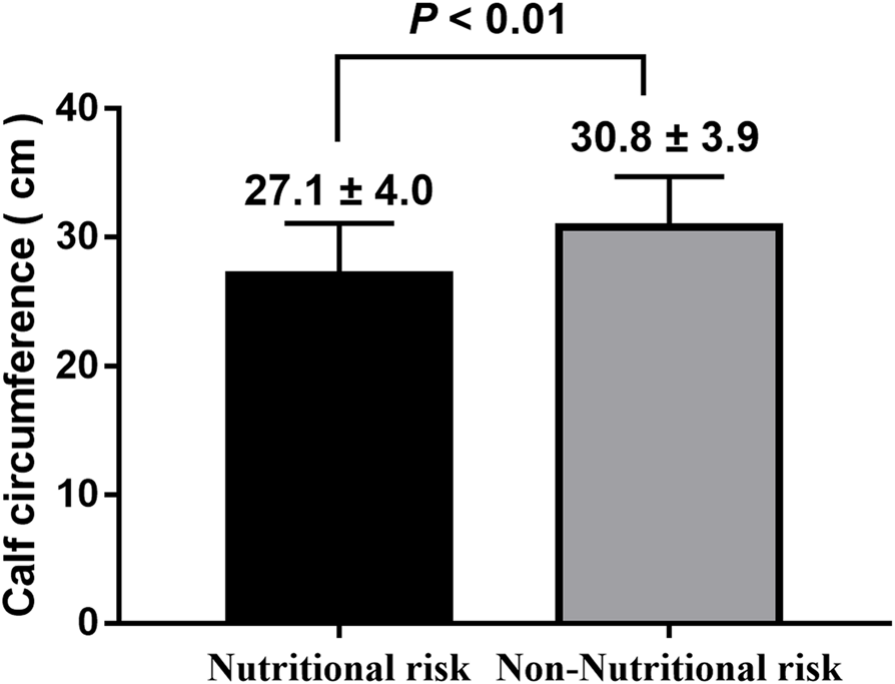
Differences in calf circumference between nutritional risk and non-nutritional risk participants using NRS 2002 diagnosis criteria

A total of 224 cases were divided as nutritional risk according to NRS 2002. The CC level was lower in the nutritional risk group than in the non-nutritional risk group (27.1 ± 4.0 cm vs. 30.8 ± 3.9 cm, *P* <  0.01) (Fig. 1).

### Factors associated with CC

CC was significantly negatively correlated with age (r = − 0.26, *P* <  0.01) and nutritional risk (r = − 0.43, *P* <  0.01). CC values were lower in males than in females (r = − 0.21, *P* <  0.01). However, CC was significantly positively related to SBP, albumin, haemoglobin, BMI and WC (all *P* <  0.01) (Table 2).

**Table 2:** Correlation analysis of clinical and biochemical parameters with calf circumference

Variable	*r*	*P*
Age^#^	−0.26	< 0.01
Male^#^	−0.21	< 0.01
Smoking status	0.02	0.63
Drinking status	0.06	0.21
SBP^#^	0.13	< 0.01
DBP	0.04	0.37
HbA1c	0.01	0.13
FPG	−0.11	0.06
TC	0.08	0.19
TG	0.06	0.27
LDL-c	0.06	0.32
HDL-c	0.22	0.71
Albumin^#^	0.31	< 0.01
Haemoglobin^#^	0.32	< 0.01
BMI^#^	0.55	< 0.01
WC^#^	0.54	< 0.01
Nutritional risk^#^	−0.43	< 0.01

BMI, body mass index; DBP, diastolic blood pressure; FPG, fasting plasma glucose; HbA1c,Haemoglobin A1c; HDL-c, high-density lipoprotein cholesterol; LDL-c, low-density lipoprotein cholesterol; SBP, systolic blood pressure; TC, total cholesterol; TG, triglyceride; WC, waist circumference

^#^*p* < 0.05

### Factors associated with nutritional risk

**Fig. 2:**
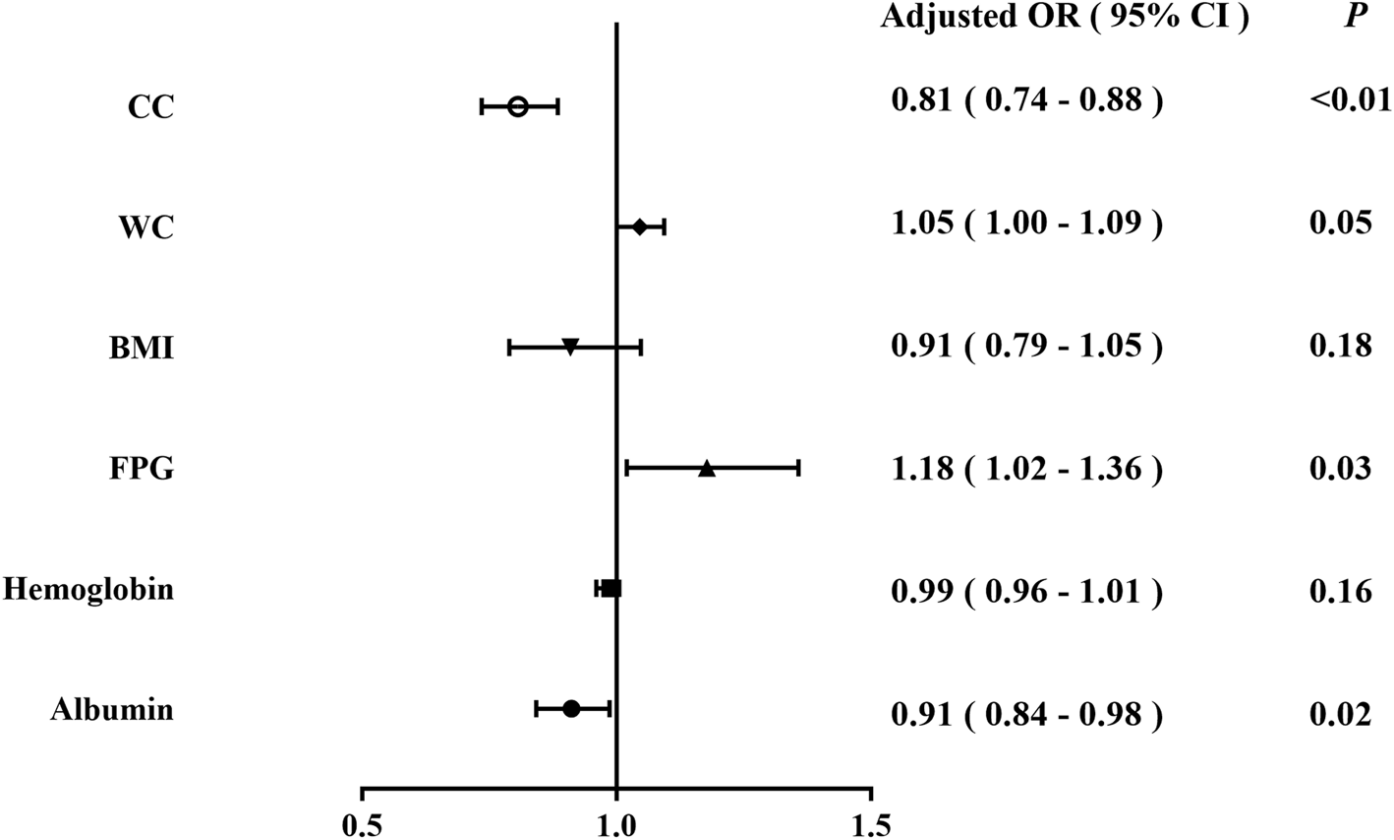
Independent factors for nutritional risk by multivariable logistic regression analysis. BMI, Body mass index; CC, Calf circumference; WC, Waist circumference. The model was adjusted for albumin, haemoglobin, FPG, BMI, WC and CC

The adjusted OR (95% CI) of CC associated with nutritional risk was calculated in the logistic regression model (Fig.2). Increasing CC (adjusted OR, 0.81; 95% CI, 0.74–0.88; *P* <  0.01) and albumin (adjusted OR, 0.91; 95% CI, 0.84–0.98; *P* = 0.02) were independent protective factors against nutrition risk. It was also revealed that increasing FPG (adjusted OR, 1.18; 95% CI, 1.02–1.36; *P* = 0.03) was an independent risk factor for malnutrition risk.

### The difference in the prevalence of nutritional risk according to the CC tertile

**Fig. 3:**
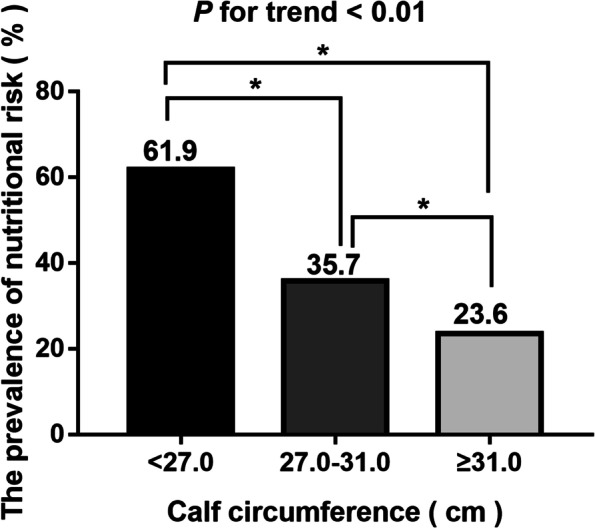
The difference prevalence of nutritional risk according to CC tertile. * *P <* 0.01

The prevalence of nutrition risk was compared according to the CC tertile. Compared with patients in tertile 3 (≥ 31.0 cm), the prevalence of nutritional risk was significantly lower among participants in tertile 2 (27.0–31.0 cm) and tertile 1 (27.0 cm) (*P* for trend < 0.01) (Fig. 3).

### The cut-off value of CC for nutritional risk

**Fig. 4:**
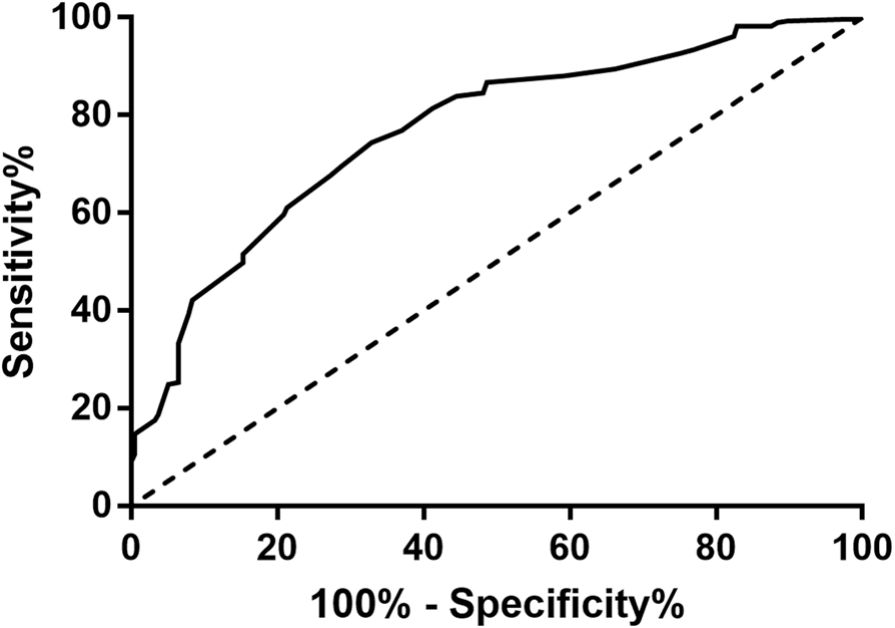
ROC curve analysis of CC for nutritional risk AUC = 0.75 (*P* < 0.01); 95% CI, 0.70–0.79; CC cut-off point = 28.8 cm; Youden index = 0.40; sensitivity, 72.0%; specificity, 67.0%

ROC curve analysis was used to find CC’s best cut-off value for identifying nutritional risk in older MetS patients. The best CC cut-off value was 28.8 cm, and the AUC was 0.75 (95% CI, 0.70–0.79), with 72.0% sensitivity and 67.0% specificity according to the Youden index (*P* <  0.01) (Fig. 4).

### Cox proportional Hazard analysis for mortality

Univariate Cox regression analysis indicated that age, SBP, DBP, FPG, albumin, haemoglobin, BMI, cerebral infarction, coronary heart disease, nutritional status and decreased CC were significantly correlated with mortality (Table 3). There were so few people who drank alcohol that we excluded drinking factors in the Cox regression analysis. All significant factors in the univariate Cox analysis were entered into the multivariate regression analysis. Finally, FPG, nutritional status, haemoglobin and decreased CC were independent factors influencing the mortality of MetS patients aged over 80 years. The risk of mortality increased 18.6% when FPG increased by 1 mmol/L. Nutritional risk (HR, 2.48; 95% CI, 1.22–5.04; *P* = 0.01) and decreased CC (HR, 2.78; 95% CI, 1.27–5.98; *P* = 0.01) remained independent risk factors for mortality. However, the risk of mortality decreased 4.4% as haemoglobin increased by 1 g/dL (Table 3).

**Table 3:** Multivariate Cox proportional hazard regression analysis of mortality in MetS

Variable	Univariate	Multivariate
	HR (95% CI) *P*	HR (95% CI) *P*
Age	1.04 (1.01–1.08) 0.03	___
Male	1.41 (0.94–2.11) 0.10	
Smoking status	1.06 (0.65–1.72) 0.82	
SBP	0.99 (0.98–0.99) < 0.01	___
DBP	0.98 (0.96–0.99) 0.04	___
HbA1c	0.90 (0.61–1.25) 0.54	
FPG	1.19 (1.01–1.30) < 0.01	1.19 (1.05–1.34) 0.01
TC	1.10 (0.85–1.42) 0.48	
TG	0.86 (0.60–1.25) 0.44	
LDL-c	1.23 (0.92–1.76) 0.15	
HDL-c	0.44 (0.13–1.54) 0.20	
Albumin	0.85 (0.82–0.88) < 0.01	___
Haemoglobin	0.95 (0.94–0.96) < 0.01	0.96 (0.94–0.97) < 0.01
BMI	0.93 (0.88–0.98) 0.01	___
WC	0.98 (0.96–1.00) 0.45	
Cerebral infarction	1.96 (1.29–2.97) < 0.01	___
Coronary heart disease	1.40 (0.89–2.19) 0.14	
Nutritional risk	4.36 (2.80–6.80) < 0.01	2.48 (1.22–5.04) 0.01
Decreased CC	2.54 (1.68–3.83) < 0.01	2.78 (1.27–5.98) 0.01

BMI, body mass index; DBP, diastolic blood pressure; FPG, fasting plasma glucose; HbA1c,Haemoglobin A1c; HDL-c, high-density lipoprotein cholesterol; LDL-c, low-density lipoprotein cholesterol; SBP, systolic blood pressure; TC, total cholesterol; TG, triglyceride; WC, waist circumference

## Discussion

The present study provided important evidence that low CC could predict nutritional risk and mortality in MetS patients aged over 80 years. The CC value was significantly lower in MetS patients with nutritional risk than in the non-nutritional risk group. The optimal CC cut-off point for predicting nutritional risk in MetS adults aged over 80 years was 28.8 cm. Subjects with CC values below 28.8 cm were defined as those with decreased CC. Moreover, decreased CC, nutritional risk and increasing FPG were independent risk factors influencing mortality in Cox regression multivariate models.

CC is a novel, useful anthropometric parameter used to measure muscle mass and is being investigated in many fields. Some studies reported that subjects with lower CC had an increased frequency of carotid plaques and was positively associated with insulin resistance and cardiovascular disease in diabetic patients [19]. In our study, we suggested that decreasing CC and increasing FPG were all independent risk factors for nutritional risk. Our previous study identified that CC is strongly related to frailty in diabetic adults aged over 80 years [23]. As geriatrics are shifting towards identifying early stages of disability, understanding frailty and sarcopenia is essential and is still developing in the quest to prevent physical dependence. Frailty and sarcopenia overlap with each other, which could bring serious health consequences, such as mobility limitations and fracture [15]. Malnutrition plays a key role in the pathogenesis of frailty and sarcopenia [24]. Malnutrition is accompanied by loss of muscle mass and muscle function and exaggerates the observed loss of fat-free mass in elderly persons, reducing metabolic reserve and insulin sensitivity [8]. Muscle plays a variety of important roles in the human body. Therefore, the loss of muscle mass and strength will lead to a variety of functional disabilities and metabolic disorders in elderly persons [25–28]. A review indicated that protein-deficient diets markedly affect skeletal muscle function, and malnourished rats present low muscle weight and impaired morphological, metabolic, and functional development. This review also showed that impaired insulin release in malnourished rats is related to lower mitochondrial oxidative and anaplerotic capacity [29].

CC was also significantly positively related to albumin, haemoglobin, BMI and WC in our study. Albumin and haemoglobin were once the standard blood biomarkers in judging nutritional status in clinical activities. It is a complex process to assess nutritional risk, including the status of recent diet and weight loss. Due to the decline of physiological function, slow movement and cognitive function, the replacement of caregivers for elderly persons, it is more challenging to assess nutritional status in elderly persons. As a simple and noninvasive index, CC can predict the risk of nutritional risk and mortality in elderly patients with MetS, which is a very significant discovery. In 2019, the Asian Sarcopenia working group recommended the use of CC for screening sarcopenia, with a critical value of < 34 cm for men and < 33 cm for women [30]. In our previous study, we concluded that the best CC cut-off value for predicting nutritional risk according to the NRS 2002 was 29.75 cm in men and 28.25 cm in women in hospitalized patients aged more than 80 years [15]. In this research, we identified the optimal CC cut-off point (28.8 cm) for predicting nutritional status in MetS adults aged over 80 years.

MetS is often accompanied by cerebral infarction and coronary heart disease, which increase mortality in elderly persons. Lim HJ showed that well-nourished status is inversely associated with cerebral infarction [31]. However, we did not find a difference in cerebral infarction or coronary heart disease between the nutritional risk and non-nutritional risk groups. Dolores Sanchez-Rodríguez also found no difference in coronary heart disease regarding nutritional status in elderly persons [32]. BMI and WC are crucial makers in defining obesity and overweight. Although low BMI is a phenotypic criterion that may lead to malnutrition diagnosis, Rocco Barazzoni showed that obesity might present with true malnutrition because of inability to maintain body composition and performance with loss of skeletal muscle loss and function that exert major negative influences on morbidity and survival [11]. However, in our study, the prevalence of abdominal obesity was lower in the nutritional risk group than in the non-nutritional risk group (46.88% vs. 65.86%), which was possibly caused by using NRS 2002 to judge nutritional status. BMI is one of the criteria in NRS 2002. Even so, the increased prevalence of nutritional risk and the decreased CC in elderly individuals with MetS were not affected. During the process of ageing, fat is redistributed from subcutaneous to abdominal depots and to liver, muscle, and other ectopic sites [33]. Our study was conducted on elderly people who were more susceptible to abdominal obesity and did not have an elevated BMI. Hence, we speculate that there may be some patients with “sarcopenic MetS” in our study population, which is a new concept. As we had expected, we found 77.2% of our population suffered from both sarcopenia and metabolic syndrome (sarcopenic MetS), which is extremely high. There may be some differences between patients with “sarcopenic MetS” and non-“sarcopenic MetS”. Further prospective studies are needed to confirm this hypothesis.

Nutritional risk was associated with a 4.4-fold increased risk of mortality after 12 years of follow-up in healthy community-dwelling French women aged 75 or older [32]. Low CC was associated with a 1.44-fold increase in mortality in patients with cancer [18]. The findings suggest the use of CC as a simple, easy, and cost-effective anthropometric measurement to quickly screen patients at risk of death. We indicated that nutritional status and decreased CC were independent risk factors in predicting the mortality of MetS. Our conclusion proved that CC might be a very useful, simple, and noninvasive index to predict nutritional status and was a perfect factor in predicting mortality in elderly individuals with MetS.

This study had some strengths. First, this study included a large sample of more than 500 subjects, which could better reflect the nutritional status of elderly patients than previous studies. Second, the longest follow-up period was 2.5 years, so we analysed the effect of nutritional status on mortality in our MetS group. Finally, we focused on subjects aged over 80 years, who are usually excluded from many studies.

This study also had some limitations. First, elderly individuals with nutritional risk defined as an NRS 2002 score ≥ 3 were supplemented, which might change the risk of mortality. Second, due to the low proportion of female subjects in this study, we were unable to estimate the potential gender impact. Finally, this is a single-centre study of inpatients in geriatric wards. All the participants in the study were Chinese, so the results may not be applicable to other ethnic groups.

## Conclusion

Decreased CC could predict not only nutritional risk but also mortality in MetS patients aged over 80 years. The optimal CC cut-off for predicting nutritional risk in our population was 28.8 cm. Our findings showed that people of very advanced age who had MetS with nutritional risk should be discovered early, and have early intervention and early treatment. CC may be a valuable index to screen out this population in MetS.

## Data Availability

The data that support the findings of this study are available from the corresponding author upon reasonable request.

## Abbreviations

BMI: Body mass index
BP: Blood pressure
CC: Calf circumference
CIs: Confidence intervals
DBP: Diastolic blood pressure
FPG: Fasting plasma glucose
HbA1c: Haemoglobin A1c
HDL-c: High-density lipoprotein cholesterol
HRs: Hazard ratios
IDF: International Diabetes Federation
LDL-c: Low-density lipoprotein cholesterol
MetS: Metabolic syndrome
NRS 2002: Nutritional Risk Screening 2002
ROC: Receiver operating characteristic
ORs: Odds ratios
SBP: Systolic blood pressure
TC: Total cholesterol
TG: Triglyceride
WC: Waist circumference
